# 

**Published:** 1858-01

**Authors:** 


### LIST OF COLLABORATORS.

S. G. Armor, M.D., Professor of Pathology and Clinical Medicine, Missouri Med. College.
John L. Atlee, M.D., President Pennsylvania State Medical Society.
Walter F. Atlee, M.D., Philadelphia.
John Bell, M.D., Philadelphia, formerly Professor of Medicine, Medical College of Ohio.
T. S. Bell, M.D., Professor of Medicine, University of Louisville.
S. M. Bemiss, M.D., Louisville, Ky.
L. P. Blackburn, M.D., Natchez, Miss.
George C. Blackie, M.D., Professor of Botany, University of Nashville.
G. C. Blackman, M.D., Professor of Surgery, Medical College of Ohio.
W. S. Bowen, M.D., New York.
J. L. Bobbs, M.D., formerly Professor of Anatomy, Indianapolis.
W. M. Boling, M.D., Montgomery, Ala., formerly Professor of Midwifery, Transylvania University, Lexington, Ky.
N. Bozeman, M.D., Montgomery, Ala.
J. T. Bradford, M.D., Augusta, Ky.
John H. Brinton, M.D., Lecturer on Operative Surgery, Philadelphia.
W. S. Chipley, M.D., Professor of Medicine, Transylvania University.
A. Clapp, M.D., New Albany, Ia.
D. B. Cliffe, M.D., Franklin, Tenn.
J. Da Costa, M.D., Lecturer on the Practice of Medicine, at the Philad. Med. Association.
M. Dupierris, M.D., Havana, Cuba.
W. F. Edgar, M.D., United States Army.
S. C. Farrar, M.D., Jackson, Miss.
C. S. Fenner, M.D., Memphis, Tenn.
Austin Flint, M.D., Professor of Clinical Medicine and Pathology, University of Buffalo.
Charles Fricke, M.D., Baltimore.
W. Y. Gadbury, M.D., Benton, Miss.
O. C. Gibbs, M.D., Chautauque County, New York.
Dr. J. P. Hall, Glasgow, Ky.
John Hardin, M.D., Professor of Obstetrics, Kentucky School of Medicine, Louisville.

EDWARD HARTSHORNE, M.D., One of the Surgeons to Wills Hospital for Diseases of the Eye, Philadelphia.

E. B. HASKINS, M.D., Clarksville, Tenn., late President of the Tennessee State Medical Society.

C. A. HENTZ, M.D., Marianna, Florida.

ADDINELL HEWSON, M.D., One of the Surgeons to Wills Hospital for Diseases of the Eye, Philadelphia.

SAMUEL L. HOLLINGSWORTH, M.D., late Editor of the Medical Examiner, Philadelphia.

WILLIAM A. HAMMOND, M.D., United States Army.

WILSON JEWELL, M.D., Philadelphia, President of the Quarantine and Sanitary Convention.

G. A. KUNKLER, M.D., Madison, Ia.

WM. V. KEATING, M.D., Lecturer on Obstetrics and the Diseases of Women, in the Philadelphia Medical Association.

BEN. LOGAN, M.D., Shreveport, La.

A. LOPEZ, M.D., Mobile, Ala., formerly President of the Alabama State Medical Society.

GEORGE R. MOREHOUSE, M.D., Philadelphia.

H. WEIR MITCHELL, M.D., Lecturer on Physiology at the Philad. Med. Association.

B. I. RAPHAEL, M.D., New York.

J. C. REEVE, M.D., Dayton, Ohio.

L. C. RIVES, M.D., formerly Professor of Midwifery, Medical College of Ohio.

J. LAWRENCE SMITH, M.D., Professor of Chemistry, University of Louisville.

W. C. SNEED, M.D., Frankfort, Ky., President Kentucky State Medical Society.

C. H. SPILMAN, M.D., Harrodsburg, Ky., late President of Kentucky State Medical Society.

GEO. SUTTON, M.D., Aurora, Ia.

W. L. SUTTON, M.D., Georgetown, Ky., formerly President Kentucky State Medical Society.

HORATIO R. STORER, M.D., formerly one of the Physicians to the Boston Lying-in Hospital, Boston, Mass.

S. THOMPSON, M.D., Albion, Ill.

E. WILLIAMS, M.D., Cincinnati, Ohio.

### Bi-Monthly Bibliographical Record.

AMERICAN.

Manual of the Botany of the Northern United States. Revised edition; including Virginia, Kentucky, and all east of the Mississippi; arranged according to the Natural System. By Asa Gray, Fisher Professor of Natural History in Harvard University. 12mo, pp. 630. Ivison & Phinney: New York, 1857. (Received from the publishers.)

First Lessons in Botany and Vegetable Physiology. Illustrated by over 300 original drawings, and furnished with a copious Glossary. By Asa Gray. 12mo, pp. 236. Ivison & Phinney: New York, 1857. (Received from the publishers.)

Materia Medica and Therapeutics; with ample illustrations of Practice, &c. &c. A new edition, revised and enlarged. By Thomas D. Mitchell, M.D., Professor of Materia Medica and Therapeutics in Jefferson Medical College, &c. 8vo, pp. 820. J. B. Lippincott & Co.: Philadelphia, 1857. (Received from the publishers.)

The Parish Will Case; Medical Opinions upon the Mental Competency of Mr. Parish. By John Watson, M.D., Pliny Earle, M.D., D. T. Browne, M.D., Luther V. Bell, M.D., M. H. Ranney, M.D., J. Ray, M.D., and Sir Henry Holland, Bart., M.D., &c. 8vo, pp. 573. J. F. Trow: New York, 1857. (Received from the authors.)

A Dictionary of Medical Science; containing a concise explanation of the various subjects and terms of Anatomy, Physiology, &c. By Robley Dunglison, M.D., &c. &c. Fifteenth edition. 8vo, pp. 992. Blanchard & Lea: Philadelphia, 1857. (Received from the publishers.)

The Principles and Practice of Obstetrics; including the Treatment of Chronic Inflammation of the Uterus, considered as a Frequent Cause of Abortion. By Henry Miller, M.D., Professor of Obstetric Medicine in the University of Louisville. 8vo, pp. 624. With illustrations. Blanchard & Lea: Philadelphia, 1857. (Received from the publishers.)

Researches on Epilepsy: its Artificial Production in Animals, and its Etiology, Nature, and Treatment in Man. Pamphlet, pp. 82. Republished from the Boston Medical and Surgical Journal. (Received from the publishers.)

Transactions of the Twelfth Annual Meeting of the Ohio State Medical Society, held in the City of Sandusky, June, 1857. 12mo, pp. 225. (From Dr. Charles Cochran, of Sandusky.)

Transactions of the American Medical Association. Vol. x. 8vo, pp. 676. Philadelphia, 1857. (Received from the Publication Committee.)

Experimental Researches relative to the Nutritive Value and Physiological Effects of Albumen, Starch, and Gum, when singly and exclusively used as Food; being one of the Prize Essays of the American Medical Association for 1857. By Wm. A. Hammond, M.D., Assistant Surgeon U. States Army, &c. &c. Pamphlet, pp. 79. Philadelphia. (Received from the author.)

Report on Infant Mortality in Large Cities, the Sources of its Increase and Means for its Diminution. By D. Meredith Rease, M.D., &c. Extracted from the Transactions of the American Medical Association. Pamphlet, pp. 19. (Received from the author.)

Essays on the Secretory and the Excito-Secretory System of Nerves in their Relations to Physiology and Pathology. By H. F. Campbell, M.D., Professor of Anatomy in Medical College of Georgia, &c. 8vo, pp. 135. J. B. Lippincott & Co.: Philadelphia, 1857. (Received from the author.)

FOREIGN.

The Science and Art of Surgery. By John Erichsen, Professor of Surgery in University College. Second edition, enlarged and revised. Illustrated by 400 engravings on wood. 8vo, pp. 1040. Walton & Maberly: London, 1857.

Pathological and Practical Observations on Diseases of the Alimentary Canal, Œsophagus, Stomach, Cœcum, and Intestines. By S. O. Habershon, M.D., &c. 8vo, pp. 387. Churchill: London, 1857.

Contributions to the Physiology and Pathology of the Circulation of the Blood. By G. Robinson, M.D., &c. 8vo, pp. 273. Longmans: London, 1857.

The Use of the Microscope in Clinical Medicine. Illustrated. By L. S. Beale, M.B., &c. &c. No. 1. Urinary Deposits. With 14 plates, containing upwards of 60 figures. 8vo, pp. 32. Churchill London, 1857.

